# Quantitative characterization of torpor-associated behaviors in mice

**DOI:** 10.1016/j.isci.2026.115590

**Published:** 2026-04-03

**Authors:** Akinobu Ohba, Hiroshi Yamaguchi

**Affiliations:** 1 Department of Cell Physiology, Nagoya University, Graduate School of Medicine, Nagoya 466-8550, Japan; 2 Division of Multicellular Circuit Dynamics, National Institute for Physiological Sciences, Okazaki, Aichi 444-8585, Japan

**Keywords:** rodent behavior, rodent physiology, physiology

## Abstract

Torpor is a hypometabolic and hypothermic state that enables small mammals such as mice to conserve energy under harsh conditions. While its physiological features have been extensively studied, behavioral signatures remain poorly characterized, partly due to limited quantitative tools. Here, we developed a posture-based framework to quantify torpor-associated behavior and test whether posture features help distinguish fasting-induced torpor from physiologically similar states. During torpor, mice actively suppress locomotion and adopt a curled-up posture with increased body width, head angle, and shortened body length as body temperature (Tb) falls. In contrast, adenosine monophosphate (AMP)-induced torpor-like state produces a stretched posture despite similarly reduced Tb, enabling robust discrimination. Using unsupervised analyses of aligned body-part coordinates, we further demonstrated that torpor-associated postural features differ from those during curled-up bouts in sleep and during cold exposure. These findings establish posture as a quantifiable behavioral marker that complements Tb and metabolic rate criteria and provide a noninvasive computational approach to refine torpor detection.

## Highlights

• Torpor is characterized by a distinctive and quantifiable curled-up posture
• Posture metrics distinguish natural torpor from AMP-induced torpor-like state
• Data-driven analysis separates torpor from similar sleep and cold postures
• Locomotion is actively suppressed before body temperature declines in fasted mice

## Graphical abstract

**Figure:**
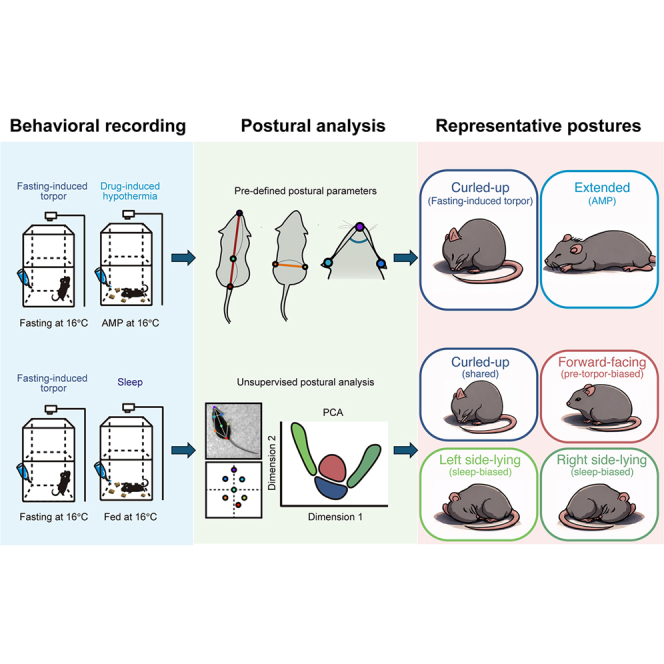

## Introduction

Maintaining a high and stable body temperature (Tb) is a key characteristic of endothermy, which supports the efficiency and stability of metabolic processes.^1^ However, this strategy requires considerable energy, particularly for small mammals and birds that lose heat quickly due to their high surface area-to-volume ratio.^2^ To conserve energy during periods of resource scarcity, many small endotherms enter a state known as torpor, characterized by a controlled reduction in metabolic rate (MR) and Tb.^3^

Torpor is widely understood as a regulated, reversible state characterized by coordinated reductions in MR and Tb, in which metabolism decreases to conserve energy. Nevertheless, in practice, many studies identify torpor using study-specific thresholds of MR and/or Tb.^3^^,^^4^^,^^5^ While this method is convenient, it is inherently arbitrary and neglects the behavioral aspects associated with torpor.^6^ This variability in MR/Tb-based operational definitions can obscure the precise onset and offset of torpor and complicate mechanistic studies, cross-species comparisons, and the development of consistent analytical frameworks.

Recent advances in computer vision and machine learning have significantly improved the way naturalistic animal behavior is measured and analyzed.^7^ Markerless pose estimation tools, such as DeepLabCut (DLC),^8^ allow researchers to track the movement of specific body parts with high spatial and temporal resolution without the need for invasive markers. These technologies have enabled the extraction of fine-grained behavioral features across a wide range of species and experimental contexts, facilitating objective, high-throughput, and quantitative behavioral analyses. Accordingly, quantitative posture metrics may complement Tb/MR-based criteria and help refine how torpor is defined and detected.

In this study, we aimed to clarify the behavioral characteristics associated with fasting-induced torpor in mice by quantitatively analyzing spontaneous activity and posture. We found that during fasting-induced torpor, mice became immobile and adopted a distinctive curled-up posture that differed from the posture observed during drug-induced torpor-like state. We then developed a posture-based analytical framework to quantify and discriminate these states. We also demonstrated that fasting-induced torpor in mice can be differentiated from other curled-up states, such as sleep and cold exposure, by using a data-driven analysis of postural features. These findings suggest that posture-based metrics offer a valuable additional approach to accurately identify fasting-induced torpor in mice.

## Results

### Tracking the behavior of mice under food deprivation in a cold environment

**Figure 1:**
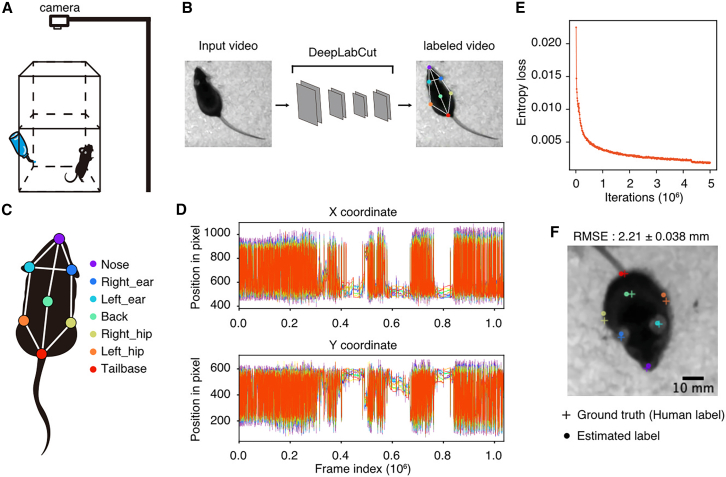
Tracking mouse behavior under food deprivation (A) A schematic setup for continuous long-term recording of a mouse during fasting. The mouse was fasted for 24 h starting from ZT12 at an ambient temperature of 16°C. A logger was intraperitoneally implanted to simultaneously measure the Tb. (B) Workflow to identify and label anatomical landmarks of mice for pose estimation using DLC. (C) Seven body parts for the pose estimation: nose (purple), right ear (dark blue), left ear (light blue), back (lime green), right hip (yellow), left hip (orange), and tailbase (red). Connecting lines represent the inferred skeletal posture. (D) A representative trace of the (x, y) coordinates in pixels for each body part over 24 h of fasting. (E) The cross-entropy loss, which quantifies the error between predictions and ground truth during training, decreased throughout 500,000 training iterations. (F) Example body part prediction for a frame that was not used during training. The average RMSE for all the test data was 2.21 ± 0.038 mm (mean ± SEM) at a p-cut off: 0.6, depending on camera height between recording sessions. Scale bars, 10 mm.

When mice are fasted from the beginning of the dark phase, they enter torpor within several hours (Figure S1).^9^^,^^10^ To investigate the behavioral alterations associated with torpor, we conducted chronic video recordings of mouse behavior with top-down acquisition while the mice fasted at 16 °C (Figure 1A). We then utilized DLC,^8^ a markerless pose-estimation software (Figure 1B), to track (x,y) coordinate changes of selected seven body parts of mice (nose, right ear, left ear, back, right hip, left hip, and tail base) (Figures 1C and 1D). The DLC model was trained for 500,000 iterations (Figure 1E), resulting in root-mean-square error (RMSE) between the true (human label) and the estimated label on the test frame of approximately 5.26 pixels (2.21 ± 0.038 mm) averaged across all key points (Figure 1F). We also visually confirmed that the trained DLC model can approximately track each body part (Video S1). Taken together, these data demonstrate that our DLC model effectively estimates and tracks behavior over 24 h.

Video S1. Representative DeepLabCut key-point predictions overlaid on raw video during fasting

### Voluntary suspension of locomotor activity upon entering torpor

**Figure 2:**
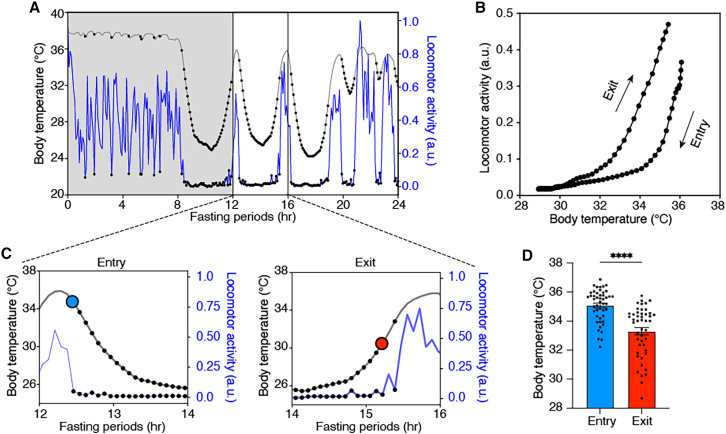
Voluntary suspension of locomotor activity associated with torpor (A) A representative trace of Tb (gray) and locomotor activity (blue) during a 24-h fasting period at 16°C. Locomotor activity is averaged over 5-min intervals and normalized to a range of 0–1. The black dots on the Tb trace and locomotor trace indicate bouts classified as immobile bouts (see Figure S2 and “locomotor analysis” in Methods). The grey-shaded area represents dark periods. (B) Relationship between Tb and locomotor activity during torpor bouts. The colored trace shows the mean across mice (each mouse trace was first averaged across its torpor bouts). Color indicates normalized time spanning from 20 min before immobility onset at torpor entry to 20 min after immobility offset at arousal. Torpor was defined as immobile bouts lasting ≥30 min with Tb < 34°C. Gray traces show the mean trajectory for each individual. (C) Enlarged representative trace of Tb and locomotor activity during torpor entry (left) and exit (right). Light blue dots represent Tb at the transition from mobile to immobile during entry, and red dots represent Tb at the transition from immobile to mobile during torpor exit. (D) Comparison of Tb during state transitions between torpor entry and exit. Bouts in which immobility lasted ≥30 min and Tb < 34°C were used as putative torpor bouts for the analysis (entry: 35.08 ± 0.16°C vs. exit: 33.30 ± 0.25°C). Data are presented as mean ± SEM. *N* = 11 mice: *n* = 47 bouts. Two-tailed Wilcoxon signed-rank test. ∗∗∗∗*p* < 0.0001.

We first analyzed the changes in locomotor activity by calculating the distance traveled by the back point. Locomotor activity is averaged over 5-min intervals and normalized to a range of 0–1 (Figure S2). Consistent with previous reports,^5^^,^^9^^,^^10^^,^^11^^,^^12^^,^^13^ we observed a marked reduction in locomotor activity as Tb decreased, often leading to prolonged immobility in all individuals (Figure 2A). Because physiological parameters (e.g., heart rate and blood pressure) in hibernating animals can change before Tb during torpor entry and exit,^14^ we analyzed transitions in Tb and locomotor activity during torpor entry and exit. To clarify the state at which mice resume movement during torpor exit, we plotted locomotor activity as a function of Tb during torpor. We found that, at comparable Tb, locomotor activity was consistently higher during exit than during entry (Figure 2B). Consistent with this, the Tb at which locomotor activity began to decrease during entry was significantly higher than the Tb at which locomotor activity began to increase during exit (entry: 35.19 ± 0.17°C vs. exit: 33.45 ± 0.26°C) (Figures 2C and 2D). These findings indicate that immobility during torpor is not simply a consequence of hypothermia. Instead, locomotion appears to be actively suppressed during the transition into torpor, as seen for other physiological parameters.^14^ Moreover, Tb begins to increase before mice resume active movement (Figure 2C), suggesting that non-shivering thermogenesis and/or shivering,^15^ rather than locomotion-induced Tb increase, play a critical role in the beginning of rewarming from torpor. Overall, these results suggest that mice actively decrease locomotion at torpor entry and resume movement after Tb partially recovers.

### Posture distinguishes fasting-induced torpor from the AMP-induced torpor-like state

**Figure 3:**
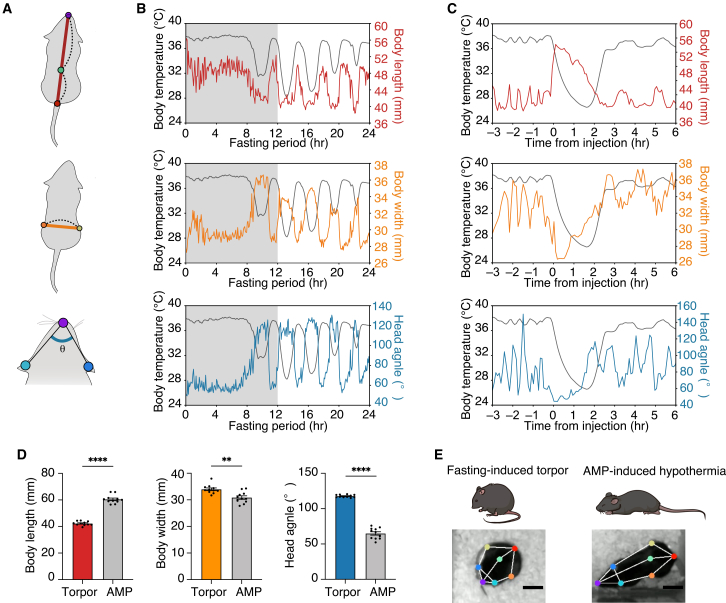
Postural distinction between torpor and AMP-induced torpor-like state (A) Extracted metrics for quantifying mouse posture: body length (top), body width (middle), and head angle (bottom). (B) A representative trace of Tb and postural metric during fasting at 16°C. The grey-shaded area represents dark periods. (C) A representative trace of Tb and postural metrics during AMP-induced torpor-like state. Each posture parameter is averaged and plotted every 5 min. (D) Comparison of the averaged postural metrics between torpor and AMP-induced torpor-like state when Tb < 34°C. Body length (Torpor: 42.32 ± 0.46 mm, AMP: ±59.09 ± 0.55 mm, Two-tailed Welch’s *t* test, *p* < 0.0001; Torpor: *n* = 11, AMP: *n* = 11), body width (Torpor: 33.99 ± 0.50 mm, AMP: 31.11 ± 0.94 mm, Two-tailed unpaired *t* test, *p* = 0.0010; Torpor: *n* = 11, AMP: *n* = 11), and head angle (Torpor: 117.75 ± 0.56°, AMP: 63.21 ± 3.05° mm, Two-tailed Welch’s *t* test, *p* < 0.0001, Torpor: *n* = 11, AMP: *n* = 11). (E) Schematic illustration and pictures showing typical posture during torpor and AMP-induced torpor-like state. Scale bars, 20 mm. Data are presented as mean ± SEM. Asterisks indicate statistical significance as follows: ∗∗*p* < 0.01 and ∗∗∗∗*p* < 0.0001.

We next conduct a quantitative analysis of postural changes associated with torpor. Based on the pixel distance between seven labeled body parts (Figure 1C), we defined three parameters to describe mouse posture: body length, body width, and head angle (Figure 3A). “Head angle” is defined as the angle at the nose formed by the segments connecting the nose to the right and left ears. This metric provides an indirect measure of lateral head tilt. When the mouse faces forward, the angle is relatively small, whereas when the head is bent downward in a curled-up posture, the angle increases. We found that these parameters changed in parallel with Tb upon fasting at 16°C (Figure 3B). Reflecting the characteristic curled-up posture during torpor, body length was shortened at lower Tb, while body width and head angle were increased (Figure 3B). These findings indicate that body length, width, and head angle are reliable parameters that effectively characterize postural changes during torpor.

While fasting is widely used to induce torpor, several pharmacological compounds have been or are currently proposed as acute inducers of artificial torpor-like states.^16^ For example, peripheral or central administration of adenosine monophosphate (AMP) has been used to induce a torpor-like hypothermic state, although the physiological equivalence to natural torpor remains debated depending on the administration route.^17^^,^^18^ However, there has been little research comparing the postural differences between the AMP-induced torpor-like state and fasting-induced torpor in mice. Therefore, we investigated the similarity of the AMP-induced torpor-like state with fasting-induced torpor in mice from a behavioral perspective. Following intraperitoneal AMP administration, mice exhibited an extended posture, characterized by increased body length (Figure 3C). This extended posture observed in AMP-induced torpor-like state contrasts with the curled-up posture typically seen during fasting-induced torpor. Quantitative comparisons of postural parameters when Tb dropped below 34 °C, one of the indicators used to define torpor,^19^ confirmed significant differences between AMP-induced torpor-like state and fasting-induced torpor (Figures 3D and 3E). Taken together, these results emphasize the effectiveness of our approach in distinguishing, in mice, fasting-induced torpor from other inducible torpor-like states that are difficult to differentiate based solely on Tb.

### Postural clustering of immobile bouts during sleep and torpor

**Figure 4:**
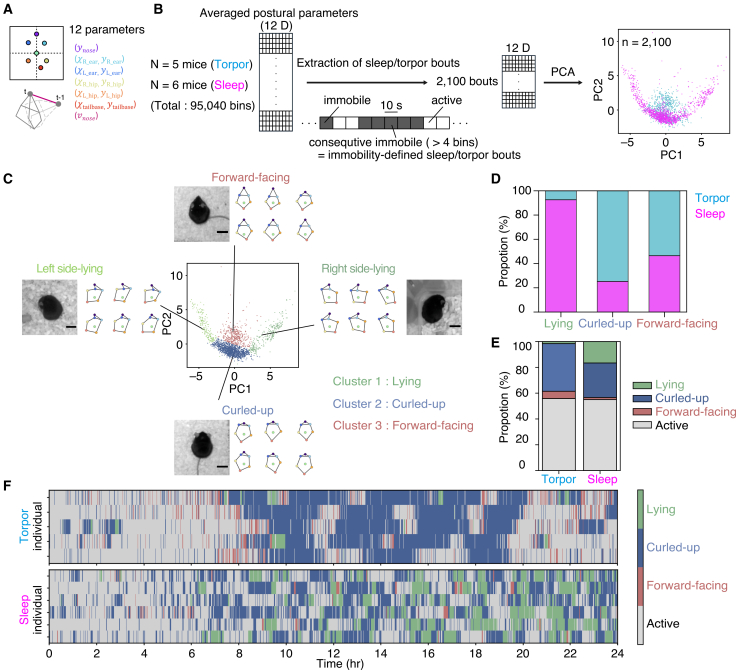
Analysis of postural features between torpor and sleep (A) Twelve postural parameters were used for the analysis (see STAR Methods). (B) Schematic of the analysis pipeline. Recorded postural data were segmented into 10-s bin either active or immobile, based on locomotor activity. Immobility-defined sleep/torpor bouts (≥4 consecutive immobile bins) were then extracted (2,100 total bouts from *N* = 5 torpor mice and *N* = 6 sleep mice). Twelve posture parameters were averaged within each bout and subjected to PCA. Two-dimensional PCA (PC1–PC2) projection of immobility-defined bouts, with each point representing a bout (bout-averaged postural features) and magenta and cyan indicating bouts from torpor and sleep recording conditions, respectively. (C) Cluster-color-coded PCA projection. Representative video frames and the corresponding body skeletons for each cluster are shown alongside the plot. Colors indicate clusters: “left-side lying” (light green), “right-side lying” (dark green), “curled-up” (blue), and “forward-facing” (red). Scale bars, 20 mm. (D) State composition of each posture cluster. Stacked bars show the proportion of bouts in each cluster from sleep (magenta) or torpor (cyan) recording condition. (E) Proportion of immobility-defined bouts assigned to each posture cluster or the active state during torpor (left) and sleep (right) recording condition. (F) Raster plot of posture-cluster and active-state sequences over time for individual animals during torpor (top) and sleep (bottom) recording conditions. Colors indicate state identity (“lying,” green; “curled-up,” blue; “forward-facing,” red; “active,” gray).

Having shown that posture analysis discriminates between fasting-induced torpor and AMP-induced torpor-like state, we next asked whether it can also distinguish torpor from sleep, another immobile state characterized by curled-up postures. To enable a direct comparison with the torpor condition, we conducted 24-h recordings in ad libitum–fed mice at the same ambient temperature (16°C; hereafter, the sleep-recording condition). This design controlled for environmental temperature, isolating the effect of fasting on posture (Figures S3A–S3C). We segmented the recording data into 10-s bins and classified each bin as active or immobile based on locomotor activity (see Materials and Methods). We operationally defined putative sleep bouts as sequences of at least four consecutive immobile bins (the “40-s rule”).^20^ All posture analyses described below were performed on these immobile bouts extracted from the sleep-recording condition and torpor recordings. Since a predefined set of simple posture metrics (e.g., body length) is insufficient to reliably distinguish curled-up postures, we adopted an unsupervised, data-driven approach that uses body-part coordinates and local movements as parameters. Specifically, we extracted a 12-dimensional set of posture features derived from aligned body-part coordinates and nose movement (see Materials and Methods) from both torpor (*n* = 5) and sleep (*n* = 6) recordings (Figure 4A). These multidimensional posture features were projected into a two-dimensional principal component analysis (PCA) space (Figure 4B), and Gaussian mixture model (GMM)-based clustering identified four distinct clusters (Figure 4C). By examining representative video frames assigned to each cluster, we annotated the clusters as right side-lying (dark green), left side-lying (light green), curled-up (blue), and facing forward (red) (Figure 4C and Video S3). Since left- and right-side lying represent essentially the same posture, we merged them into a single cluster, yielding three clusters: (1) lying, (2) curled-up, and (3) forward-facing. The curled-up cluster was present in both sleep and torpor recordings, whereas lying postures were predominantly observed during sleep recordings (Figures 4D and 4E). This suggests that postures during torpor are highly stereotyped, consistently adopting a curled-up form. In contrast, sleep comprises a more diverse repertoire of postures, including curled-up and multiple lying positions. The forward-facing posture cluster was predominantly observed during torpor recordings and was particularly frequent before the onset of Tb decrease (Figures 4F and S4). In this cluster, mice adopt a largely immobile posture accompanied by subtle head and/or nose movements, potentially reflecting sniffing and environmental scanning (Video S3). Overall, these analyses indicate that, even among immobile bouts, torpor and sleep differ in their repertoire of postures, with torpor bouts being dominated by the curled-up cluster and a distinct forward-facing cluster.

Video S3. Representative body-skeleton postures for clustered immobile posture bouts

### Postural features discriminate between torpor and sleep within the curled-up cluster

**Figure 5:**
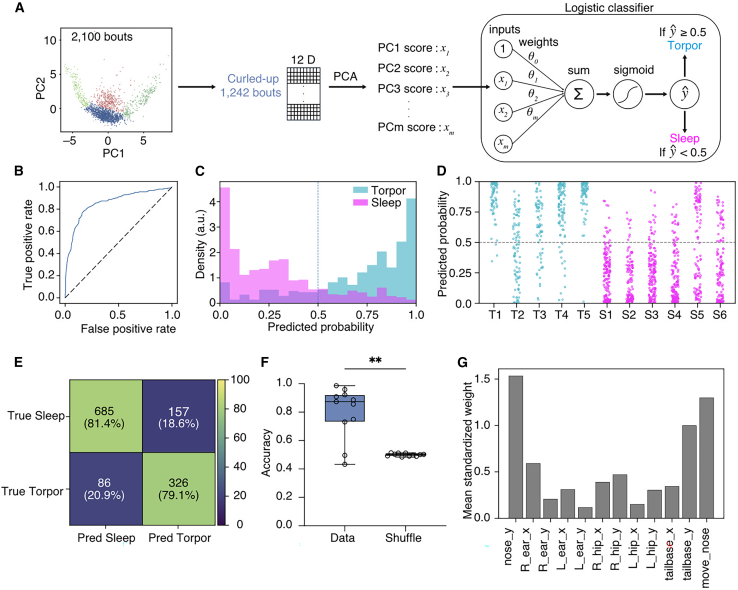
Discrimination of curled-up bouts during torpor and sleep (A) Schematic of the analysis pipeline for classifying bouts assigned to the “curled-up” cluster (Cluster 2; 1,242 bouts). Twelve-dimensional postural features were averaged within each immobility-defined sleep or torpor bout and subjected to PCA; the resulting PC scores were used as inputs to a logistic regression classifier that outputs the probability of torpor for each bout. The number of PCs was chosen such that the cumulative explained variance reached ≥90%, and PC1–PCm were used as classifier inputs. (B) ROC curve of the classifier from leave-one-mouse-out cross-validation, showing true positive rate versus false positive rate across decision thresholds. (C) Distribution of predicted torpor probabilities for torpor (cyan) and sleep (magenta) bouts; the vertical dashed line indicates the decision threshold = 0.5. (D) Predicted torpor probability for individual bouts grouped by animal (T1–T5, torpor; S1–S6, sleep). The horizontal dashed line indicates the decision threshold. (E) Confusion matrix summarizing classification performance; numbers and percentages indicate the count and proportion of bouts in each state. (F) Accuracy comparison between data and a shuffled-label control (labels were shuffled within the training set in each cross-validation fold), Two-tailed Wilcoxon matched-pairs signed rank test (*n* = 11), ∗∗*p* < 0.01). (G) Mean absolute standardized logistic regression weight for each postural feature; larger values indicate stronger contributions to discriminating torpor from sleep.

To test separability within the shared curled-up cluster, we extracted immobile bouts assigned to the curled-up cluster (*n* = 1,242 bouts) from both sleep and torpor recordings and performed PCA on the 12-dimensional posture features. We then trained a logistic regression classifier on the resulting principal components (PCs) to assign each bout a probability of belonging to the torpor class. Model performance was assessed using leave-one-mouse-out cross-validation. In each fold, the lowest-dimensional set of PCs explaining ≥90% of the variance was selected, and the corresponding PC scores were used as classifier inputs (with PCA and the classifier fitted on the training set and applied to the held-out mouse). This approach achieved robust discriminative performance (AUROC = 0.856; Figure 5B). We then classified each bout using a decision threshold of 0.5 for simplicity (Figures 5C, 5D, and S5). Overall accuracy was 80.6% (Figure 5E); the performance was significantly higher than that obtained with shuffled labels (Figure 5F). When classifier inputs were restricted to only the first two PCs (PC1 and PC2), a commonly used low-dimensional representation, classification performance decreased markedly, consistent with the substantial overlap between sleep and torpor bouts in the PC1–PC2 space (Figure S6). These results suggest that state-specific information is embedded in higher-order, lower-variance dimensions beyond PC1–PC2. Although these dimensions account for relatively little variance, they capture subtle posture differences that carry state-specific information. Analysis of model weights suggested that vertical nose position, tail-base position, and nose movement contributed most strongly to the discrimination between torpor and sleep (Figure 5F). Overall, these findings indicate that although both torpor and sleep can involve immobile curled-up postures, they represent separable states even within the same curled-up posture class.

### Postural features distinguish torpor from cold exposure

**Figure 6:**
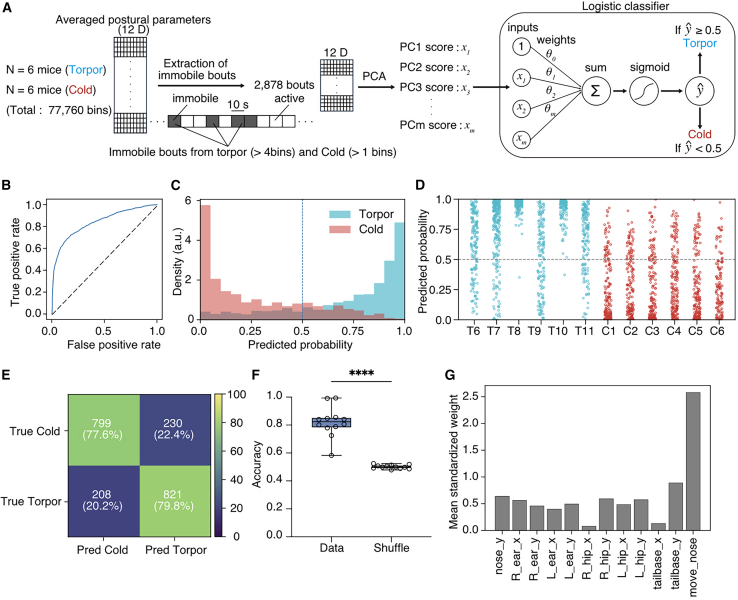
Classification of postural features between torpor and cold exposure (A) Schematic of the analysis pipeline for classifying torpor versus cold exposure. Twelve-dimensional postural parameters were segmented into 10-s bins in torpor (*N* = 6 mice) and cold-exposure (*N* = 6 mice) recordings (total bins: 77,760). Immobile bouts (2,878 bouts) were extracted and subjected to PCA, and the resulting PC scores were used as inputs to a logistic regression classifier that outputs the probability of torpor for each bin. The number of PCs was chosen such that the cumulative explained variance reached ≥90%, and PC1–PCm were used as classifier inputs. (B) ROC curve of the classifier from leave-one-mouse-out cross-validation, showing true positive rate versus false positive rate across decision thresholds. (C) Distribution of predicted torpor probabilities for torpor (cyan) and cold-exposed (red) immobile bouts; the vertical dashed line indicates the decision threshold = 0.5. (D) Predicted torpor probability for individual immobile bins grouped by animal (T6–T11, torpor; C1–C6, cold exposure). The horizontal dashed line indicates the decision threshold. (E) Confusion matrix summarizing classification performance; numbers and percentages indicate the count and proportion of bouts in each state. (F) Accuracy comparison between data and a shuffled-label control (labels were shuffled within the training set in each cross-validation fold), Two-tailed paired *t* test (*n* = 12), ∗∗∗∗*p* < 0.0001. (G) Mean absolute standardized logistic regression weight for each postural feature; larger values indicate stronger contributions to discriminating torpor from sleep.

Mice exposed to cold environments often adopt a curled posture as part of a cold-defense response to maintain Tb. We therefore asked whether postural features during fasting at 16°C can be distinguished from those during severe cold exposure. To address this, we recorded mice's behavior under cold conditions at 4 °C. To reduce confounding by sleep postures at 4 °C, we analyzed cold exposure recordings only during the 12-h dark period. For the torpor recordings, we extracted immobility-defined bouts as sequences of at least four consecutive immobile 10-s bins (≥40 s), as described above. In contrast, because curled-up postures during severe cold exposure can occur transiently, we extracted cold-exposure bouts as sequences of one or more consecutive immobile bins (≥10 s). We then combined torpor bouts and cold-exposure immobile bouts and performed PCA on the 12 postural features (torpor, *n* = 6; cold exposure, *n* = 6), projecting the bouts into a two-dimensional PC space (Figure 6A). Unlike the distinct clustering observed between torpor and sleep in the 2D PCA map, torpor bouts and cold bouts did not form clearly separable clusters (Figure S7). Next, we constructed a logistic regression classifier to distinguish torpor from cold exposure using the 12 posture features extracted from all immobile bouts, following the same approach used for the torpor–sleep classification. Model performance was assessed using leave-one-mouse-out cross-validation, achieving robust discriminative performance (AUROC = 0.880; Figure 6B). Bouts were then classified using a decision threshold of 0.5 (Figures 6C, 6D, and S7). Overall accuracy was 78.9%, with 79.8% of cold exposure bouts and 77.6% of torpor bouts correctly classified (Figure 6E). This performance was significantly higher than that obtained with shuffled labels (Figure 6F). Analysis of model weights indicated that nose movement contributed most strongly to discriminating torpor from cold exposure (Figure 6G), potentially reflecting cold-induced increases in feeding-associated head movements, along with more frequent changes in body orientation. These results indicate that, despite their superficial similarity, torpor and cold exposure can be discriminated by postural features.

## Discussion

In this study, we quantitatively characterized behaviors associated with fasting-induced torpor. We found that mice progressively reduced locomotion before core Tb began to decline, indicating that torpor entry is preceded by detectable changes in locomotion. We also showed that postural features carry state-specific information that can discriminate torpor from a drug-induced torpor-like state or superficially similar curled-up states observed during sleep and cold exposure. Together, these findings establish posture as a robust behavioral marker of fasting-induced torpor in mice that complements other physiological readouts.

Our analysis effectively distinguished fasting-induced torpor from the AMP-induced torpor-like state.^17^ Although both states involve reduced Tb, peritoneally AMP-injected mice did not adopt the curled-up posture characteristic of natural torpor; instead, they maintained an extended body posture. This postural difference may reflect the distinct physiological profiles associated with different administration routes. While the central administration of adenosine receptor agonists has been reported to induce coordinated metabolic suppression resembling natural torpor,^21^^,^^22^ peripheral administration has been suggested to differ from natural torpor.^23^^,^^24^ These postural differences between physiologically similar hypothermic states underscore the utility of posture as a sensitive behavioral marker that refines torpor detection.

We found that mice exhibited subtle head or nose movements just before Tb decline in torpor recordings. This pattern corresponds to the forward-facing posture cluster identified in our unsupervised clustering. Because these behaviors were rarely observed in sleep recordings, they likely reflect an awake and vigilant state rather than sleep. These behaviors may represent a transitional phase in which mice balance foraging demands with energetic constraints before entering torpor. Supporting this idea, we have previously shown that inhibitory neurons in the dorsomedial hypothalamus (DMH^Vgat^), which are indispensable for torpor entry during fasting, become active before Tb begins to decline.^9^ Activating these neurons does not induce torpor when mice are fed. This suggests that these neurons may play a role in a preparatory transitional phase rather than directly causing torpor. This pre-torpor behavioral change is also consistent with other state transitions, such as sleep, in which observable behavioral changes precede detectable alterations in the electroencephalogram.^25^^,^^26^^,^^27^ Therefore, our behavioral data suggest a preparatory phase for torpor that is not merely a result of hypothermia but rather reflects active regulation by specific neural systems.

Current definitions of torpor often depend on Tb or metabolic thresholds.^3^^,^^4^^,^^5^ However, torpor is not a uniform phenomenon. Even within the same individual, the depth and duration of torpor bouts can vary. Additionally, certain mouse strains and individuals exhibit relatively shallow torpor, characterized by only modest decreases in Tb.^28^^,^^29^^,^^30^^,^^31^ In our dataset, we occasionally observed shallow drops in Tb immediately after the onset of fasting. Our classifier variably labeled these shallow episodes as either “torpor” or “sleep,” depending on the bout (Figure S5). This suggests that postural features are insufficient to clearly determine whether these brief, shallow hypothermic episodes represent torpor or sleep. Future work integrating posture-based analysis with additional physiological readouts (e.g., EEG/EMG, tail temperature, or metabolic measurements) may enable more accurate discrimination of such shallow hypothermic episodes. Nonetheless, our findings support incorporating behavioral metrics into the operational definition of torpor, which enables more comprehensive and inclusive detection across diverse physiological conditions and genetic backgrounds.

Activating several specific neuronal populations in the preoptic nucleus can induce a torpor-like hypothermic state. These neuronal groups include adenylate cyclase-activating polypeptide 1 (Adcyap1),^10^ pyroglutamylated RFamide peptide (QRFP),^32^ estrogen receptor α (ERα),^33^ transient receptor potential melastatin 2 (TRPM2),^9^ and prostaglandin EP3 receptor (EP3R)^34^ neurons. However, the artificial states they produce may not fully recapitulate energy conservation, such as increased locomotor activity or the absence of a curled-up posture typically seen in natural torpor. Our posture-based framework provides a scalable and biologically grounded approach to address this gap by offering a quantitative, behavior-based measure of how closely induced hypometabolic/hypothermic torpor-like states resemble natural torpor at the behavioral level.

### Limitations of the study

We acknowledge the limitation that a single frame of a torpor-like curled-up posture cannot reliably distinguish torpor from other similar states. In addition, our use of a single camera view limited our ability to detect specific features, such as tail movements, which may also serve as behavioral markers of torpor. Utilizing improved imaging techniques, including multi-angle or 3-dimensional reconstruction approaches,^35^^,^^36^^,^^37^^,^^38^ is likely to enhance the resolution and accuracy of posture-based detection of torpor. Nevertheless, our results support the idea that postural features provide useful cues for discriminating torpor from other similar states.

In conclusion, we introduce a noninvasive, behavior-based framework for detecting torpor in mice that captures behavioral signatures near its onset and distinguishes it from drug-induced hypothermic states. This approach offers a refined, high-resolution tool for studying hypometabolic behaviors in laboratory animals and may facilitate future investigations into the underlying neural circuits and adaptive significance of torpor. The classifier and methodology we present can be extended to other physiological states and species, allowing for broader applications in behavioral neuroscience and related biomedical research.

## Resource availability

### Lead contact

Further information and requests for resources should be directed to and will be fulfilled by the lead contact, Hiroshi Yamaguchi (yamaguch@nips.ac.jp).

### Materials availability

This study did not generate new unique reagents.

### Data and code availability

• Data: Data reported in this paper will be shared by the lead contact upon request.
• Code: All analyses were performed using custom code written in Python. Code generation and debugging were assisted by the large language model ChatGPT (OpenAI). All original code has been deposited at GitHub and is publicly available as of the date of publication. DOI is https://zenodo.org/records/19228747.
• Any additional information: Any additional information required to reanalyze the data reported in this paper is available from the lead contact upon request.

## Acknowledgments

This work was supported by the 10.13039/501100001691Japan Society for the Promotion of Science 10.13039/501100001691KAKENHI to H.Y. (23K26830, 24H02007, 25K22336), to A.O. (23H04939), the 10.13039/501100002241Japan Science and Technology Agency 10.13039/501100009023PRESTO to H.Y. (JPMJPR21SA), 10.13039/100007449Takeda Science Foundation, 10.13039/501100012036Lotte Foundation, and 10.13039/501100005694Hori Sciences and Arts Foundation to H.Y.

## Author contributions

A.O. and H.Y. designed the study. A.O. developed methodology, performed experiments, analyzed data, and created figures. A.O. and H.Y. wrote the manuscript. H.Y. supervised the project.

## Declaration of interests

The authors declare no conflicts of interest.

## STAR★Methods

### Key resources table

**Table:** 

REAGENT or RESOURCE	SOURCE	IDENTIFIER
**Chemicals, peptides, and recombinant proteins**		

Adenosine-5′ monophosphate sodium salt	Nacalai	01748–71

**Deposited data**		

Codes	This paper	https://zenodo.org/records/19228747

**Experimental models: Organisms/strains**		

Mouse: C57BL/6J	clea Japan	C57BL/6JJcl

**Software and algorithms**		

Adobe Creative Cloud(Illustrator)	Adobe	https://creativecloud.adobe.com/cc
Prism 9	GraphPad Software	https://www.graphpad.com/
Mercury	Star-oddi	https://www.star-oddi.com/products/accessories/mercury--application-software
DeepLabCut (v2.3.9)	Mackenzie Weygandt Mathis lab	https://sourceforge.net/projects/deeplabcut.mirror/files/v2.3.9/

**Other**		

ultra-small temperature logger	Star-Odd	DST Nano-T
ELP 2-MP USB camera	Shenzhen Ailipu Technology Co., Ltd.	ELP-USBFHD08S-MFV(5-50 mm)-J

### Experimental model and study participant details

All C57BL/6J mice (13–15 weeks old) were housed under a 12-h light/dark cycle at an ambient temperature of ∼22°C, with *ad libitum* access to standard chow and water. All experimental procedures were approved by the Animal Care and Use Committee of Nagoya University (Approval No.: R240004) and the National Institute for Physiological Sciences (Approval No.: 25A065). Efforts were made to minimize the number of animals used and their suffering.

### Method details

#### Body temperature measurements

Core Tb was recorded using intraperitoneally implanted loggers (Star-Oddi, DST nano-T) under isoflurane anesthesia. Experiments were performed after a recovery period of at least 7 days. Tb data were recorded every 5 min using Mercury (Star-Oddi) from ZT12 on the experiment day to ZT12 the following day (289 data points). Averaging over each 5-min window yielded 288 bins for analysis. To plot the cluster or predicted states color band against Tb trace in Figures S3, S5, and S7, Tb data were linearly interpolated at 10-s intervals to align with the bin length.

#### Induction of torpor and AMP-induced torpor-like state

For torpor induction, mice were placed into transparent square cages (259 × 234 × 209 mm) (Allentown Inc) with bedding at ZT12, maintained at 16 °C, and fasted for 24 h with *ad libitum* access to water. For the pharmacologically induced torpor-like state, mice were placed at ZT12 into the square cages at either room temperature (*n* = 5) or 16°C (*n* = 6) with *ad libitum* access to food and water, and at ZT15 received an intraperitoneal injection of AMP (400 mg/kg; Nacalai) dissolved in saline. For sleep recording, mice were placed at ZT12 into the square cages at 16 °C for 24 h with *ad libitum* access to water. For cold exposure, mice were placed into the square cages at ZT12 for 12 h inside a temperature-controlled chamber preset to 4 °C (SHIN-FACTORY, HC-100).

#### Video recording

Top-view video recordings were made using an ELP 2-MP USB camera (Shenzhen Ailipu Technology Co., Ltd.) mounted on a tension rod. Red LED illumination was employed during the dark phase of recording. The videos were captured at a frame rate of 12 fps in grayscale, with a resolution of 1280 × 720, for 24 h (torpor and sleep) or 12 h (AMP-induced torpor-like state and cold exposure). Mice that failed to rewarm spontaneously for 24 h fasting were exclusively used for training the DeepLabCut model and were excluded from further analysis. Video and logger recordings were synchronized by starting both from the same Windows PC, with time-limited recording sessions lasting either 24 or 12 h beginning at ZT12.

#### Pose estimation with DeepLabCut

DeepLabCut (v2.3.9) was used for markerless pose estimation. From 11 randomly selected videos (torpor and AMP conditions), 100 frames per video were extracted using K-means clustering. The extracted frames were split into 75% for training and 25% for testing. Seven body parts (nose, right ear, left ear, back, right hip, left hip, and tail base) were manually annotated in the training frames. A ResNet-50–based network was trained for 500,000 iterations, with snapshots saved every 10,000 iterations. The snapshot that achieved the lowest root-mean-square error (RMSE) on the test frames was used for subsequent analyses. The same training and tracking procedure were applied to the sleep and cold-exposure video sets. The training was carried out on an Intel Core i9-12900HX with 64 GB of RAM and an NVIDIA GeForce RTX 4060 Laptop GPU with GDDR6 8 GB (Mouse Computer Co., Ltd.) running Windows 11.

#### Extraction of pre-defined postural metrics

Extraction of postural metrics was performed on the tracked data using custom-written Python code. Locomotion was calculated from the Euclidean distance between successive positions of the “back” point using the following formula:

$$\text{Locomotion}=\sqrt{{\left(x_{\text{back}}\left(t-1\right)-x_{\text{nose}}\left(t\right)\right)}^{2}+{\left(y_{\text{back}}\left(t-1\right)-y_{\text{back}}\left(t\right)\right)}^{2}}$$

#### Four postural features were calculated as follows

$$\text{Body}\;\text{length}=\sqrt{{\left(x_{\text{tail}}-x_{\text{nose}}\right)}^{2}+{\left(y_{\text{tail}}-y_{\text{nose}}\right)}^{2}}$$

$$\text{Body}\;\text{width}=\sqrt{{\left(x_{\text{right}\_\text{hip}}-x_{\text{left}\_\text{hip}}\right)}^{2}+{\left(y_{\text{right}\_\text{hip}}-y_{\text{left}\_\text{hip}}\right)}^{2}}$$

The angle between two vectors defined by three points (p1, p2, p3) was calculated as follows: Define vectors $\vec{v1}$ and $\vec{v2}$ from point p2 to p1 and from p2 to p3, respectively:

$$\vec{v1}=\left(x1-x2,y1-y2\right),\vec{v2}=\left(x3-x2,y3-y2\right)$$

Normalize these vectors to obtain unit vectors unit_v1 and unit_v2, compute the angle from the dot product using the arc cosine function,

$$\text{uni}t_{v1}=\frac{\vec{v1}}{\left\|\vec{v1}\right\|},\text{uni}t_{v2}=\frac{\vec{v2}}{\left\|\vec{v2}\right\|}$$

$$\text{angle}\_\text{rad}=\arccos\left(\text{uni}t_{v1}・\text{uni}t_{v2}\right)$$

$$\text{angle}=\text{angle}\_\text{rad}\times \frac{180}{\pi }$$

For the head angle, the points (p_right_ear, p_nose, p_left_ear) were used, and for the body bend angle, the points (p_nose, p_back, p_tailbase) were used to perform these calculations. To correct slight height variations between recording sessions, we scaled the recordings based on the on-screen cage size.

#### Locomotor analysis

To detect long-lasting immobile bouts during fasting, 12 Hz locomotion data over 24 h were averaged into 5-min bins (288 points per animal). A Gaussian kernel density estimation (KDE) with 1,000 points was applied to histograms of locomotor activity:

$$f\left(x\right)=\frac{1}{n}\sum_{i=1}^{n}K_{h}\left(x-x_{i}\right)$$

where K_h_ is the Gaussian kernel, and h is a bandwidth estimated by Scott’s rule. This procedure produced a smooth locomotion distribution that typically exhibited a bimodal pattern, corresponding to immobile and mobile states. We defined the activity threshold as the inflection point near the lower-activity peak, identified from the derivative of the KDE. Each 5-min bin was then classified as mobile or immobile using this threshold. Thresholds were computed independently for each mouse. To quantify the relationship between locomotor activity and Tb around torpor entry and exit (Figure 2B), torpor bouts were identified as immobile periods that (i) lasted at least 30 min and (ii) reached Tb < 34 °C. For each bout, a peri-bout window spanning 20 min before bout onset to 20 min after bout offset was extracted. Within this window, each bout was aligned to a normalized 0–1 timescale. Locomotor activity and Tb traces were averaged across bouts within each mouse, mean traces were obtained by averaging the individual trace.

#### Postural discrimination analysis in torpor and other curled-up state

To characterize postural features during torpor and other curled-up states, an unsupervised, data-driven approach based on body-part coordinates and a local movement metric was used. First, the (x, y) coordinates of seven tracked body points were extracted (14 parameters). To exclude information about absolute position in the cage and heading direction relative to the cage walls (upper vs. lower wall), the coordinates were horizontally shifted so that the back point served as the origin (thus, *x* back = 0 and *y* back = 0) and were rotated to align the body axis toward the upper wall (thus, *x* nose = 0). This alignment yielded 11 postural features. In addition, to capture subtle local movements, frame-to-frame changes in nose position were computed and included as an additional feature, yielding a 12-dimensional postural feature set.

#### Postural clustering in immobility-defined torpor and sleep

Recordings of postural data were segmented into 10-s bins, and each bin was classified as active or immobile. In this analysis, immobile bins were defined as those in which the cumulative pixel displacement of the back point was <50 pixels within a 10-s bin. To detect sleep bouts, consecutive immobile bins lasting ≥40 s (≥4 bins) were grouped into immobility bouts; these bouts were labeled as sleep (*ad libitum* feeding) or torpor (fasting) based on the experimental condition. For each bout, a mean posture feature vector was computed by averaging the 12 postural features across all bins belonging to that bout. Before dimensionality reduction, the bout-averaged 12 features were standardized across bouts. PCA was then applied to the standardized feature matrix, and bouts were visualized in the space of the first two principal components (PC1–PC2). Finally, to identify postural clusters while avoiding over-segmentation, we fit a Gaussian mixture model (GMM; k = 4) to the PCA representation.

#### Logistic regression classification

For torpor versus sleep classification, all bouts assigned to the curled-up cluster were extracted from both the sleep- and torpor-recording conditions. For torpor versus cold exposure classification, all immobility-defined bouts were extracted from both the cold exposure and torpor recording conditions, with bout definitions matched to each condition (cold exposure: consecutive immobile bins ≥1; torpor: consecutive immobile bins ≥4). For each bout, twelve-dimensional postural features were averaged across all bins belonging to the bout and subjected to PCA; the resulting PC scores were used as inputs to a binary logistic regression classifier trained to discriminate torpor from the comparison state (sleep or cold exposure). The number of PCs was set to the smallest value such that the cumulative explained variance reached ≥90%, and PC1–PCm were used as classifier inputs. Leave-one-animal-out cross-validation was performed with an L2-regularized logistic regression model (default C = 1.0), and class imbalance was corrected within each training fold by random undersampling of the majority class such that the two classes contributed equally to model fitting. Standardization, PCA, and logistic regression were fitted using only the training data in each fold and were then applied to the corresponding held-out animal. Bouts were classified using a fixed decision threshold of 0.5 on the predicted torpor probability. Classification performance was quantified by accuracy and compared against a shuffled-label control in which bout labels were randomly permuted within each training fold, and fold-averaged accuracy was computed across 100 shuffles. At this decision threshold, a confusion matrix was generated, and standard performance metrics (accuracy, sensitivity, specificity, precision, and negative predictive value) were computed. To interpret model coefficients in terms of the postural features, logistic-regression weights were summarized as follows. In each cross-validation fold, the classifier was trained on PC scores, and coefficients were therefore obtained in PC space. These coefficients were then back-projected to the standardized 12-dimensional postural-feature space using the PCA loading matrix estimated from the training data in the same fold. To obtain a robust, sign-independent measure of contribution, the absolute value of each back-projected standardized coefficient was computed within each fold and averaged across folds. The resulting mean absolute standardized weights were visualized as a bar plot (“Mean standardized weight”), with larger values indicating features that more consistently contributed to the linear decision boundary across animals under the PCA-based representation.

### Quantification and statistical analysis

All data were presented as the mean ± standard error of the mean (SEM). Statistical analyses were performed using GraphPad Prism version 9.0.2 for macOS (GraphPad, USA). A *p*-value less than 0.05 was considered statistically significant. To test normality, the Shapiro–Wilk test was performed.

## Supplemental information

Document S1. Figures S1–S7

Video S2. Frame-by-frame extraction of behavioral metrics using custom Python code

## References

[1] Clarke A., Pörtner H.-O. (2010). Temperature, metabolic power and the evolution of endothermy. Biol. Rev. Camb. Phil. Soc..

[2] Reitman M.L. (2018). Of mice and men - environmental temperature, body temperature, and treatment of obesity. FEBS Lett..

[3] Ruf T., Geiser F. (2015). Daily torpor and hibernation in birds and mammals. Biol. Rev. Camb. Phil. Soc..

[4] Hudson J.W., Scott I.M. (1979). Daily Torpor in the Laboratory Mouse, Mus musculus Var. Albino. Physiol. Zool..

[5] Sunagawa G.A., Takahashi M. (2016). Hypometabolism during Daily Torpor in Mice is Dominated by Reduction in the Sensitivity of the Thermoregulatory System. Sci. Rep..

[6] Geiser F., Breed M., Moor J. (2010). Encyclopedia of Animal Behavior.

[7] Egnor S.E.R., Branson K. (2016). Computational analysis of behavior. Annu. Rev. Neurosci..

[8] Mathis A., Mamidanna P., Cury K.M., Abe T., Murthy V.N., Mathis M.W., Bethge M. (2018). DeepLabCut: markerless pose estimation of user-defined body parts with deep learning. Nat. Neurosci..

[9] Yamaguchi H., Murphy K.R., Fukatsu N., Sato K., Yamanaka A., de Lecea L. (2023). Dorsomedial and preoptic hypothalamic circuits control torpor. Curr. Biol..

[10] Hrvatin S., Sun S., Wilcox O.F., Yao H., Lavin-Peter A.J., Cicconet M., Assad E.G., Palmer M.E., Aronson S., Banks A.S. (2020). Neurons that regulate mouse torpor. Nature.

[11] Swoap S.J., Gutilla M.J. (2009). Cardiovascular changes during daily torpor in the laboratory mouse. Am. J. Physiol. Regul. Integr. Comp. Physiol..

[12] Williams T.D., Chambers J.B., Henderson R.P., Rashotte M.E., Overton J.M. (2002). Cardiovascular responses to caloric restriction and thermoneutrality in C57BL/6J mice. Am. J. Physiol. Regul. Integr. Comp. Physiol..

[13] Ambler M., Hitrec T., Wilson A., Cerri M., Pickering A. (2022). Neurons in the Dorsomedial Hypothalamus Promote, Prolong, and Deepen Torpor in the Mouse. J. Neurosci..

[14] Horwitz B.A., Chau S.M., Hamilton J.S., Song C., Gorgone J., Saenz M., Horowitz J.M., Chen C.Y. (2013). Temporal relationships of blood pressure, heart rate, baroreflex function, and body temperature change over a hibernation bout in Syrian hamsters. Am. J. Physiol. Regul. Integr. Comp. Physiol..

[15] Fons R., Sender S., Peters T., Jürgens K.D. (1997). Rates of rewarming, heart and respiratory rates and their significance for oxygen transport during arousal from torpor in the smallest mammal, the Etruscan shrew Suncus etruscus. J. Exp. Biol..

[16] Bouma H.R., Verhaag E.M., Otis J.P., Heldmaier G., Swoap S.J., Strijkstra A.M., Henning R.H., Carey H.V. (2012). Induction of torpor: Mimicking natural metabolic suppression for biomedical applications. J. Cell. Physiol..

[17] Zhang J., Kaasik K., Blackburn M.R., Lee C.C. (2006). Constant darkness is a circadian metabolic signal in mammals. Nature.

[18] Jinka T.R., Tøien Ø., Drew K.L. (2011). Season primes the brain in an arctic hibernator to facilitate entrance into torpor mediated by adenosine A(1) receptors. J. Neurosci..

[19] Pack A.I., Galante R.J., Maislin G., Cater J., Metaxas D., Lu S., Zhang L., Von Smith R., Kay T., Lian J. (2007). Novel method for high-throughput phenotyping of sleep in mice. Physiol. Genom..

[20] Swoap S.J., Rathvon M., Gutilla M. (2007). AMP does not induce torpor. Am. J. Physiol. Regul. Integr. Comp. Physiol..

[21] Tupone D., Madden C.J., Morrison S.F. (2013). Central activation of the A1 adenosine receptor (A1AR) induces a hypothermic, torpor-like state in the rat. J. Neurosci..

[22] Morrison S.F., Cano G., Hernan S.L., Chiavetta P., Tupone D. (2025). Inhibition of the hypothalamic ventromedial periventricular area activates a dynorphin pathway-dependent thermoregulatory inversion in rats. Curr. Biol..

[23] Iliff B.W., Swoap S.J. (2012). Central adenosine receptor signaling is necessary for daily torpor in mice. Am. J. Physiol. Regul. Integr. Comp. Physiol..

[24] Strijkstra A.M., Koopmans T., Bouma H.R., de Boer S.F., Hut R.A., Boerema A.S., Ruf T., Bieber C., Arnold W., Millesi E. (2012). Living in a Seasonal World.

[25] Sotelo M.I., Tyan J., Markunas C., Sulaman B.A., Horwitz L., Lee H., Morrow J.G., Rothschild G., Duan B., Eban-Rothschild A. (2022). Lateral hypothalamic neuronal ensembles regulate pre-sleep nest-building behavior. Curr. Biol..

[26] Tossell K., Yu X., Giannos P., Anuncibay Soto B., Nollet M., Yustos R., Miracca G., Vicente M., Miao A., Hsieh B. (2023). Somatostatin neurons in prefrontal cortex initiate sleep-preparatory behavior and sleep via the preoptic and lateral hypothalamus. Nat. Neurosci..

[27] Eban-Rothschild A., Rothschild G., Giardino W.J., Jones J.R., de Lecea L. (2016). VTA dopaminergic neurons regulate ethologically relevant sleep-wake behaviors. Nat. Neurosci..

[28] Suita K., Ishikawa K., Kaneko M., Wataki A., Takahashi M., Kiyonari H., Sunagawa G.A. (2023). Mouse embryonic stem cells embody organismal-level cold resistance. Cell Rep..

[29] Nowack J., Mzilikazi N., Dausmann K.H. (2023). Saving energy via short and shallow torpor bouts. J. Therm. Biol..

[30] Song X., Körtner G., Geiser F. (1997). Thermal relations of metabolic rate reduction in a hibernating marsupial. Am. J. Physiol. Regul. Integr. Comp. Physiol..

[31] Reher S., Dausmann K.H. (2021). Tropical bats counter heat by combining torpor with adaptive hyperthermia. Proc. Biol. Sci..

[32] Takahashi T.M., Sunagawa G.A., Soya S., Abe M., Sakurai K., Ishikawa K., Yanagisawa M., Hama H., Hasegawa E., Miyawaki A. (2020). A discrete neuronal circuit induces a hibernation-like state in rodents. Nature.

[33] Zhang Z., Reis F.M.C.V., He Y., Park J.W., DiVittorio J.R., Sivakumar N., van Veen J.E., Maesta-Pereira S., Shum M., Nichols I. (2020). Estrogen-sensitive medial preoptic area neurons coordinate torpor in mice. Nat. Commun..

[34] Machado N.L., Lynch N., Costa L.H., Melville D., Kucukdereli H., Kaur S., Banks A.S., Raffin F., Ramirez-Plascencia O.D., Aten S. (2025). Preoptic EP3R neurons constitute a two-way switch for fever and torpor. Nature.

[35] Nath T., Mathis A., Chen A.C., Patel A., Bethge M., Mathis M.W. (2019). Using DeepLabCut for 3D markerless pose estimation across species and behaviors. Nat. Protoc..

[36] Karashchuk P., Rupp K.L., Dickinson E.S., Walling-Bell S., Sanders E., Azim E., Brunton B.W., Tuthill J.C. (2021). Anipose: A toolkit for robust markerless 3D pose estimation. Cell Rep..

[37] Hong W., Kennedy A., Burgos-Artizzu X.P., Zelikowsky M., Navonne S.G., Perona P., Anderson D.J. (2015). Automated measurement of mouse social behaviors using depth sensing, video tracking, and machine learning. Proc. Natl. Acad. Sci. USA.

[38] Wiltschko A.B., Johnson M.J., Iurilli G., Peterson R.E., Katon J.M., Pashkovski S.L., Abraira V.E., Adams R.P., Datta S.R. (2015). Mapping sub-second structure in mouse behavior. Neuron.

