# Supplementary material for: Quantitative characterization of torpor-associated behaviors in mice
Source: iScience. 2026 Apr 3;29(5):115590. doi: 10.1016/j.isci.2026.115590 (PMC13101675; doi:10.1016/j.isci.2026.115590)
Supplement: Document S1. Figures S1–S7 [file mmc1.pdf]

iScience, Volume *29*

Supplemental information

Quantitative characterization

# of torpor-associated behaviors in mice

Akinobu Ohba and Hiroshi Yamaguchi

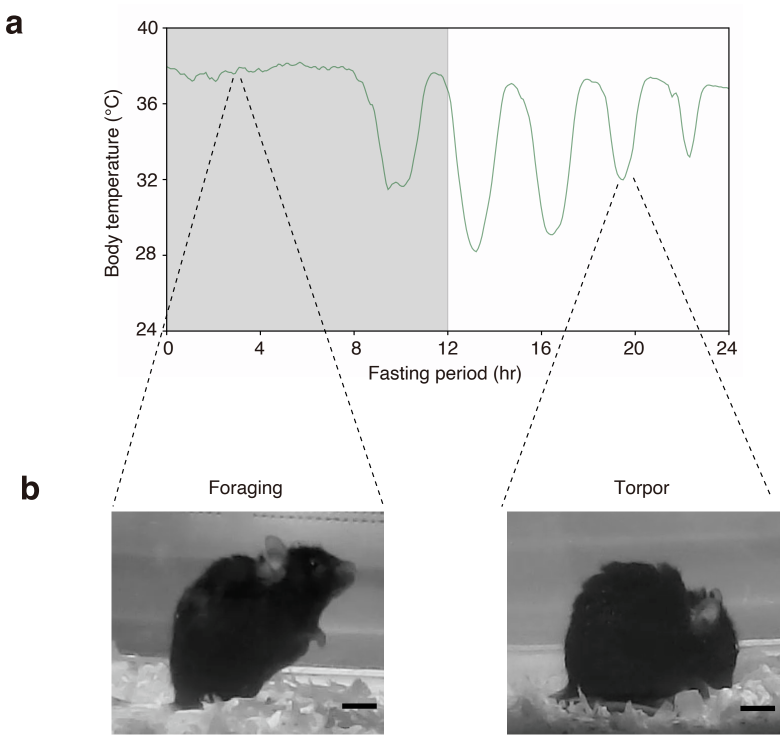

Figure S1. Mice exhibited distinctive curled-up posture during torpor.

(**a**) Representative Tb changes in the mouse fasted for 24 hours at 16°C. Greyed areas indicate dark periods. (**b**) Representative pictures of the mouse during foraging and torpor. Scale bar, 10 mm.

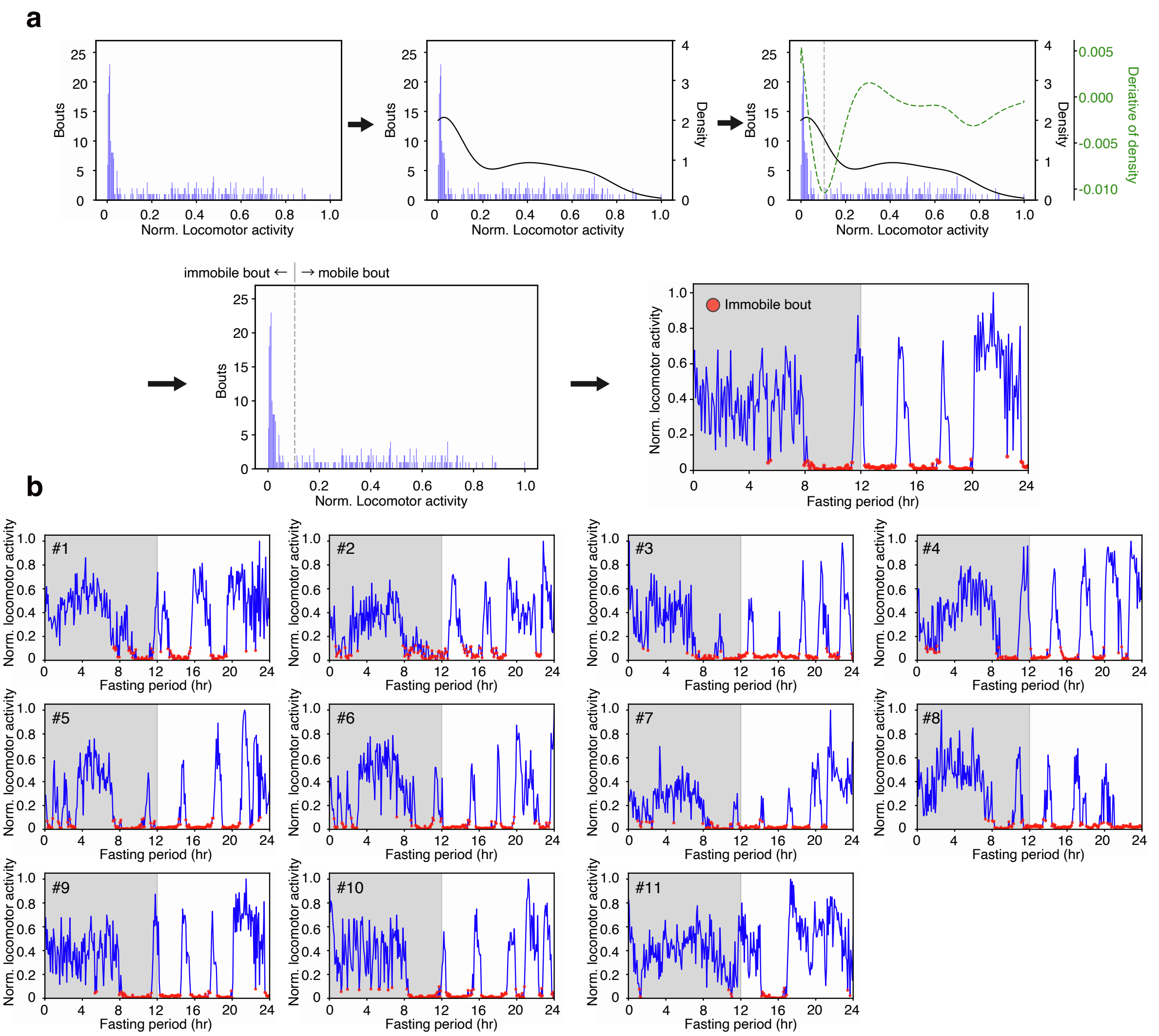

Figure S2. Classification of mobile and immobile states.

(**a**) Pipeline for threshold setting: Locomotor activity data were divided into 5-minute bins by averaging, normalized to a range of 0 to 1, and used to generate a histogram. Gaussian kernel density estimation (KDE) was subsequently applied to produce a smooth distribution which typically exhibited a bimodal pattern (black line). The inflection points of KDE determined from a derivative of density (dashed green line) at the lower locomotor peak were used as the threshold (dashed grey line) for classifying each bin into mobile or immobile states. Thresholds were calculated individually for each mouse. (**b**) Visualization of immobile bouts in all mice. The grey-shaded area represents dark periods. Red dots represent bouts classified as immobile.

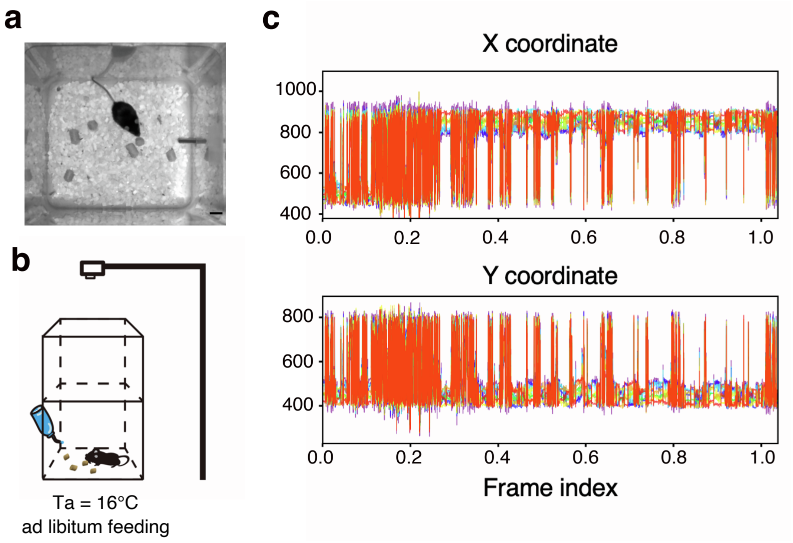

Figure S3. Tracking mouse behavior under ad libitum feeding at 16°C for sleep recordings.

(**a**) A representative frame for the sleep recordings. Scale bar, 20 mm. (**b**) A schematic setup for continuous long-term recording of mouse for sleep posture analysis. Mice were continuously recorded for 24 hours starting at ZT12 under ad libitum feeding. (**c**) A representative trace of the (x, y) coordinates in pixels for each body part over 24 hours recording.

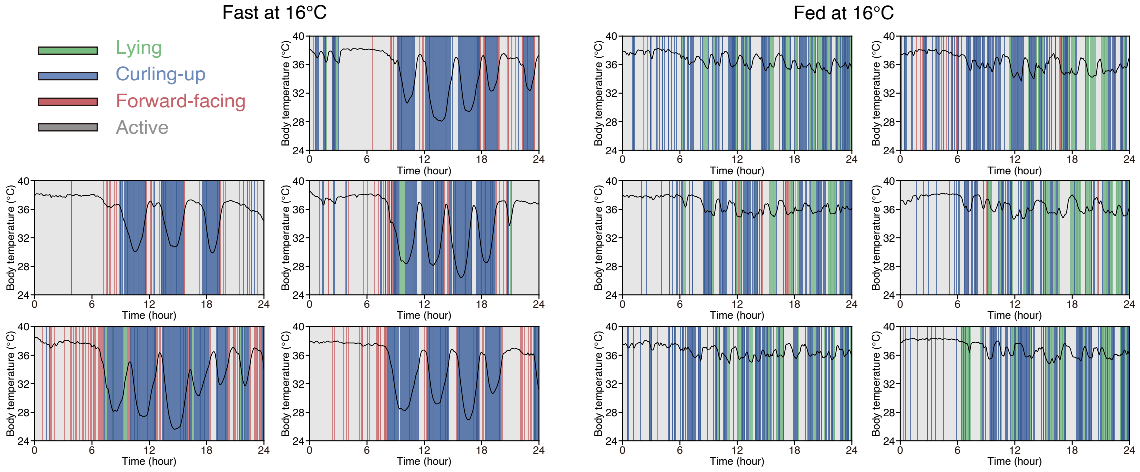

Figure S4. Overlay of clustered immobile posture bouts and body temperature traces.

Individual data are shown for torpor mice (left, n = 5) and sleep mice (right, n = 6). Black lines indicate Tb traces, and colored bands represent immobility-defined bouts assigned to each posture cluster: Lying (green), Curled-up (blue), and Forward-facing (red). Active bins are shown in gray.

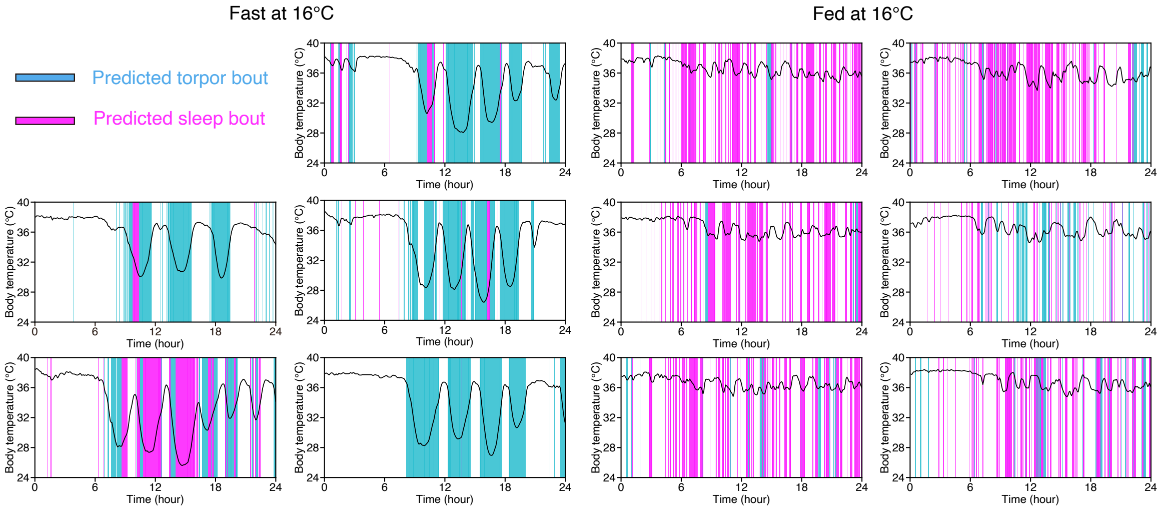

Figure S5. Overlay of predicted sleep/torpor states and body temperature traces.

Individual data are shown for torpor mice (left, n = 5) and sleep mice (right, n = 6). Black lines indicate Tb traces, and colored bands represent immobility-defined bouts classified as torpor (cyan) or sleep (magenta).

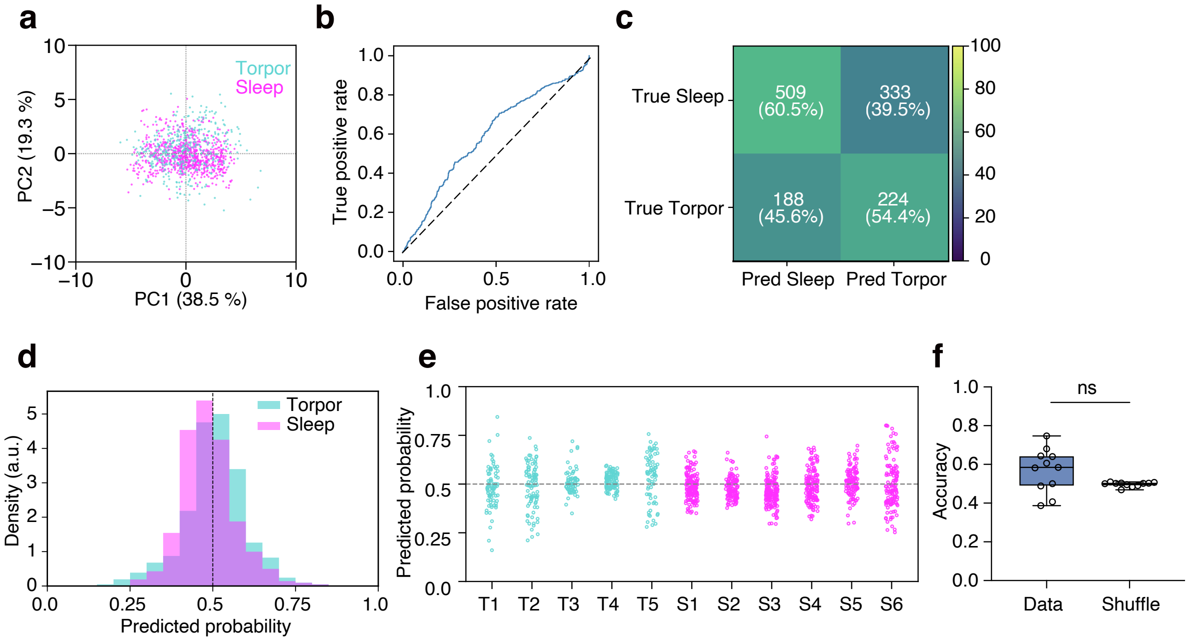

Figure S6. Classification performance for distinguishing sleep versus torpor curled-up bouts using only PC1–PC2.

(**a**) Two-dimensional PCA map (PC1 vs PC2) of curled-up bouts, color-coded by state (torpor, cyan; sleep, magenta). (**b**) ROC curve for the PC1–PC2-based logistic regression classifier. (**c**) Confusion matrix summarizing classification performance; numbers and percentages indicate the count and proportion of bouts in each state. (**d**) Distribution of predicted torpor probabilities for torpor (cyan) and sleep (magenta) bouts; the vertical dashed line indicates the decision threshold = 0.5. (**e**) Predicted torpor probability for individual bouts grouped by animal (T1–T5, torpor; S1–S6, sleep). The horizontal dashed line indicates the decision threshold. (**f**) Accuracy comparison between data and a shuffled-label control (labels were shuffled within the training set in each cross-validation fold), Two-tailed Wilcoxon matched-pairs signed rank test (n = 11), p = 0.1230; ns, not significant.

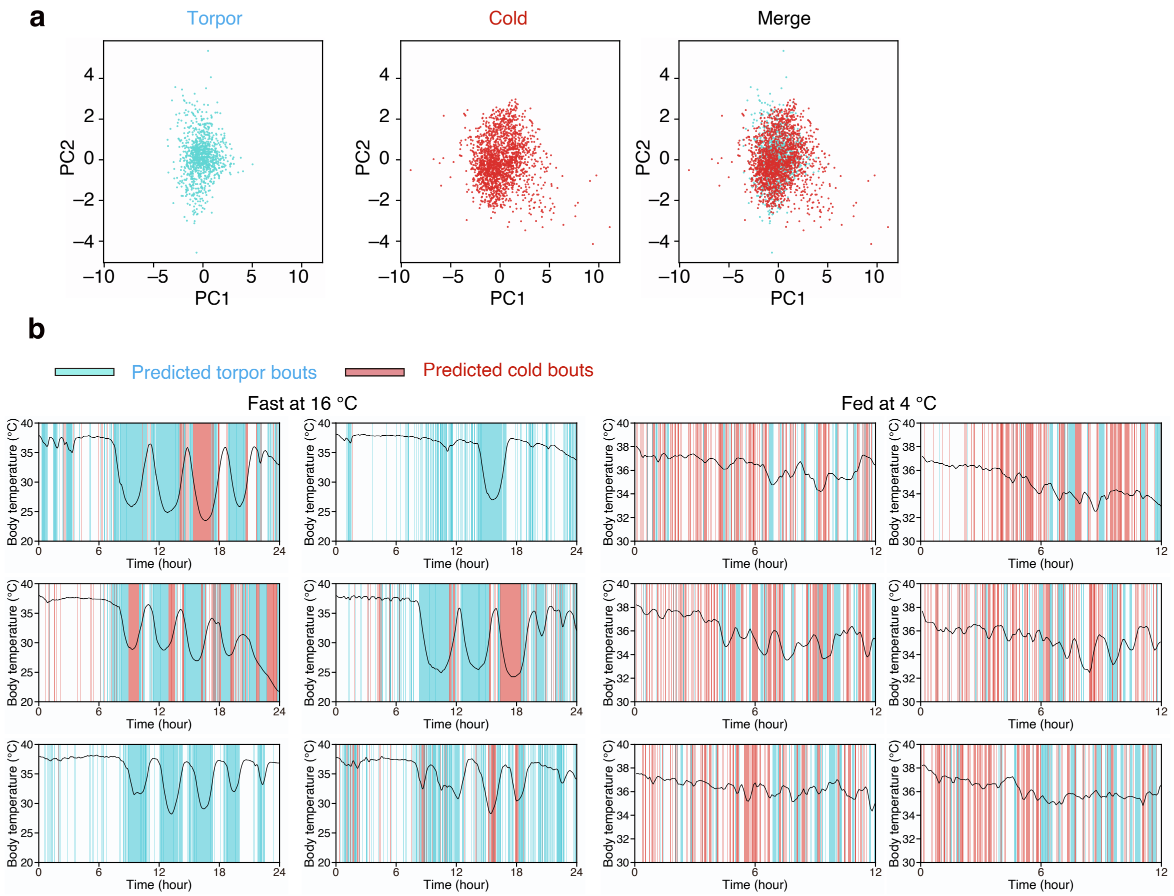

Figure S7. Detailed analysis of posture-based classification between torpor and cold exposure.

(**a**) Two-dimensional PCA map (PC1–PC2) of immobile bouts from torpor (cyan) and cold exposure (red) recording condition. (**b**) Individual data are shown for torpor mice (left, n = 6) and cold-exposed mice (right, n = 6). Black lines indicate Tb traces, and colored bands represent immobile bouts classified as torpor (cyan) or cold exposure (red).
